# Tau-PET imaging in Parkinson's disease: a systematic review and meta-analysis

**DOI:** 10.3389/fneur.2023.1145939

**Published:** 2023-04-27

**Authors:** Junjiao Zhang, Jianing Jin, Dongning Su, Tao Feng, Huiqing Zhao

**Affiliations:** ^1^Department of Neurology, Center for Movement Disorders, Beijing Tiantan Hospital, Capital Medical University, Beijing, China; ^2^China National Clinical Research Center for Neurological Diseases, Beijing, China; ^3^Department of Neurology, Beijing Tiantan Hospital, Capital Medical University, Beijing, China

**Keywords:** Parkinson's disease, cognitive impairment, neurodegenerative diseases, tau, PET, meta-analysis

## Abstract

**Background:**

Pathological tau accumulates in the cerebral cortex of Parkinson's disease (PD), resulting in cognitive deterioration. Positron emission tomography (PET) can be used for *in vivo* imaging of tau protein. Therefore, we conducted a systematic review and meta-analysis of tau protein burden in PD cognitive impairment (PDCI), PD dementia (PDD), and other neurodegenerative diseases and explored the potential of the tau PET tracer as a biomarker for the diagnosis of PDCI.

**Methods:**

PubMed, Embase, the Cochrane Library, and Web of Science databases were systematically searched for studies published till 1 June 2022 that used PET imaging to detect tau burden in the brains of PD patients. Standardized mean differences (SMDs) of tau tracer uptake were calculated using random effects models. Subgroup analysis based on the type of tau tracers, meta-regression, and sensitivity analysis was conducted.

**Results:**

A total of 15 eligible studies were included in the meta-analysis. PDCI patients (*n* = 109) had a significantly higher tau tracer uptake in the inferior temporal lobe than healthy controls (HCs) (*n* = 237) and had a higher tau tracer uptake in the entorhinal region than PD with normal cognition (PDNC) patients (*n* = 61). Compared with progressive supranuclear palsy (PSP) patients (*n* = 215), PD patients (*n* = 178) had decreased tau tracer uptake in the midbrain, subthalamic nucleus, globus pallidus, cerebellar deep white matter, thalamus, striatum, substantia nigra, dentate nucleus, red nucleus, putamen, and frontal lobe. Tau tracer uptake values of PD patients (*n* = 178) were lower than those of patients with Alzheimer's disease (AD) (*n* = 122) in the frontal lobe and occipital lobe and lower than those in patients with dementia with Lewy bodies (DLB) (*n* = 55) in the occipital lobe and infratemporal lobe.

**Conclusion:**

*In vivo* imaging studies with PET could reveal region-specific binding patterns of the tau tracer in PD patients and help in the differential diagnosis of PD from other neurodegenerative diseases.

**Systematic review registration:**

https://www.crd.york.ac.uk/PROSPERO/.

## 1. Introduction

Parkinson's disease (PD) is a common neurodegenerative disorder, pathologically characterized by the presence of α-synuclein (α-Syn)-rich Lewy bodies (1). However, accumulating evidence suggests that tau protein, which is generally associated with tauopathies such as Alzheimer's disease (AD), progressive supranuclear palsy (PSP), and argyrophilic grain disease, is also involved in the pathophysiology of PD (2, 3). The role of tau protein in PD development may be through crosstalk with α-Syn, resulting in the loss of physiological function and axonal transport dysfunction, ultimately leading to the deposition of toxic fibrils and cell death (2). Some studies have found abnormal deposition of tau in the brains of PD dementia (PDD) and PD mild cognitive impairment (PD-MCI) patients (4, 5), suggesting that it may be related to the cognitive impairment of PD patients. The effect of tau protein on PD patients is related to the site of deposition. Therefore, the *in vivo* visualization of tau protein deposition in PD patients is required.

With the tau tracer, positron emission tomography (PET) can visualize and quantify the tau burden in the brain *in vivo* (6, 7). The first tau-PET study specifically for PD patients reported that the tau tracer uptake value in the lateral temporal lobe of PD without dementia (PDND) patients was lower than that of PDD (8). There are more tau-PET studies for PD patients since then, but the region-specific pattern of tau burden varied across studies. It is related to the type and stage of PD patients, especially the cognitive status of PD patients. These results could also be influenced by the binding properties of the tau tracer. Therefore, it is necessary to investigate and compare the tau deposition in the brain of PD patients with and without cognitive impairment as shown by different tracers.

There are two aims of our meta-analysis. First, we investigated the binding patterns of the tau-PET tracer in PD patients with different cognitive statuses, including PD with uncertain cognitive status, PD with normal cognition (PDNC), PD with cognitive impairment (PDCI), PDND (PDNC and PD-MCI were included), and PDD. We explored the potential of tau radiotracers as biomarkers for PDCI. Second, we compared the differences in tau burden in the brains of PD patients and healthy controls (HCs) or other neurodegenerative diseases (PSP, AD, dementia with Lewy bodies [DLB], multiple system atrophy—parkinsonian type [MSA-P], and multiple system atrophy—cerebellar type [MSA-C]).

## 2. Methods

The meta-analysis was designed and implemented according to the Preferred Reporting Items for Systematic Reviews and Meta-Analyses (PRISMA) guidelines. We registered this meta-analysis on PROSPERO (CRD42022330981).

### 2.1. Search strategy

A literature search was performed in PubMed, Embase, the Cochrane Library, and Web of Science databases up to 1 June 2022. The retrieval process used the strategy of combining MeSH terms and free words, which were developed under the theme of positron emission tomography, tau, and Parkinson's disease. The full electronic search strings are outlined in Supplementary material (Supplementary Table 1). In addition, to ensure a comprehensive search, we also conducted a manual search of the references to the retrieved articles.

### 2.2. Study selection

The titles and abstracts were screened independently by two reviewers (JZ and JJ). Studies meeting the following criteria were included: (a) case–control study or longitudinal study; (b) subjects included PD patients diagnosed according to the UK Brain Bank criteria (9) or the Movement Disorder Society (MDS) criteria (10); (c) tau tracer PET measures reported sufficient information to calculate effect sizes; (d) studies that did not include HCs were also included in the meta-analysis but only for PD subgroup analysis; and (e) studies written in English and published in a peer-reviewed journal. If multiple studies evaluated the same sample, we included the study with the largest sample size. Exclusion criteria were as follows: (a) reviews, editorials, comments, and animal experiments and (b) case reports or small sample size studies (sample size is <5).

### 2.3. Quality assessment

The quality of the included studies was assessed by the Newcastle–Ottawa Scale (NOS) criteria (11), which included selection (0–4 scores), comparability (0–2 scores), and exposure (0–3 scores). The score ranged from 0 to 9, with not <7 indicating high research quality. Disagreement was resolved by consensus and by seeking for opinion of a third reviewer (TF).

### 2.4. Data extraction

Two reviewers (JZ and JJ) independently extracted the required data. We extracted the following information from each study: (a) information about study characteristics (author's first name and year of publication); (b) patient characteristics (number of participants, mean age, sex ratio [female %], disease duration [year], Unified Parkinson's Disease Rating Scale [UPDRS] Part-III motor score, Mini-Mental State Examination [MMSE] score, and Montreal Cognitive Assessment [MoCA] score); and (c) PET imaging aspects (tracer used for PET, tau tracer uptake value of various brain regions, and analysis methods). If PET measurements were reported as median and range (min–max), we used an online calculator to transform the data (http://www.math.hkbu.edu.hk/~tongt/papers/median2mean.html). For qualitative results reported graphically, we obtained quantitative results through GetData software.

### 2.5. Statistical analysis

Data were analyzed using Stata 16.0 software (STATA Corporation, College Station, TX). Standardized mean differences (SMDs) and 95% confidence intervals (95% CI) between patients with PD (including PD with uncertain cognitive status, PDNC, PDCI, and PDD) and HCs or other neurodegenerative diseases were calculated using the random-effects model. Meta-analysis was conducted for results reported by at least two independent studies in each brain region. We used *I*^2^ to evaluate heterogeneity, and an *I*^2^ higher than 50% was considered indicative of significant study heterogeneity (12).

Subgroup analyses were performed according to the generation of PET tracers (first- and second-generation tracers) and the type of PET tracers to further explore the source of heterogeneity. In our meta-analysis, the first-generation tracers included ^18^F-FDDNP, ^18^F-AV-1451, and ^18^F-THK-5351 and the second-generation tracers included ^18^F-PI-2620 and ^18^F-APN-1607.

Sensitivity analyses were used to assess the stability of the results by omitting one study in turn and recomputing the pooled estimates for the remaining studies. Meta-regression analyses were planned to explore the relationship between tau burden and age, disease duration, UPDRS motor score, and MMSE score when at least 10 studies were available for each modifier. Egger's test for funnel plot asymmetry was used to investigate the possibility of publication bias (13). Publication bias was verified by the cut-and-fill method. The statistical significance was set at a *p* < 0.05 two-tailed.

## 3. Results

### 3.1. Literature search

**Figure 1:**
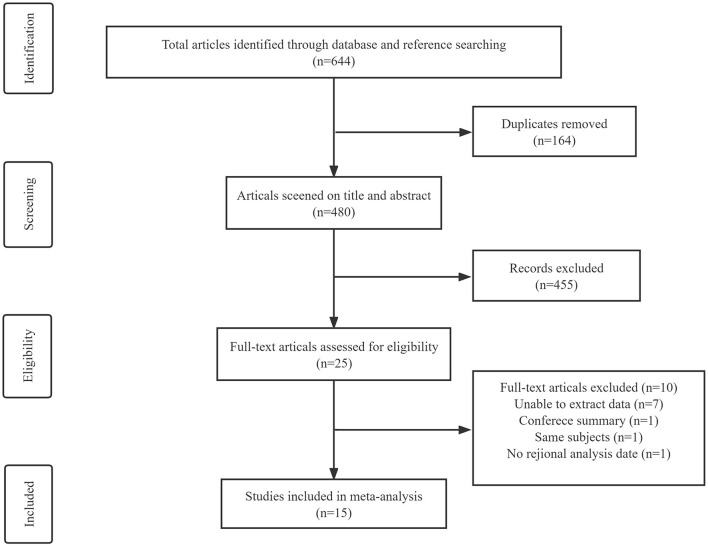
Flow diagram of the study selection process.

A comprehensive search in PubMed, Embase, Web of Science, and the Cochrane Library databases yielded 644 articles. After removing 164 duplicate articles, the remaining articles were screened by title and abstract. A total of 25 articles were selected for full-text screening, of which seven had unobtainable data, one was a conference abstract, one had no data on the brain regions of interest, and one article was an overlapping study published by the same author. A total of 15 studies (8, 14–27) were finally included for meta-analysis (Figure 1), including 90 PD (the cognitive status was not determined), 109 PDCI, 26 PDD, 61 PDNC, 27 PDND, 364 HCs, 215 PSP, 122 AD, 55 DLB, 16 MSA-P, and 6 MSA-C.

### 3.2. Characteristics of included studies

The general characteristics of included studies were shown in Table 1. A total of five tau tracers were used in the included studies (^18^F-AV-1451, ^18^F-FDDNP, ^18^F-THK-5351, ^18^F-PI-2620, and ^18^F-APN-1607). The first-generation tracers included ^18^F-AV-1451, ^18^F-FDDNP, and ^18^F-THK-5351 and the second-generation tracers included 18F-PI-2620 and 18F-APN-1607. There are 10 studies using ^18^F-AV-1451; two studies using ^18^F-FDDNP tracer; and three studies using ^18^F-THK-5351, ^18^F-PI-2620, and ^18^F-APN-1607 tracers, respectively. In total, 13 included studies were of high research quality. The remaining two studies did not include community controls, and the comparability of the cases and controls was poor, with a NOS score of 6, indicating a relatively low research quality (Supplementary Table 2).

**Table 1:** Characteristics of included studies.

**References**	**Tracer**	**Outcome**	**Subjects**									**Age**	**Male (%)**	**Duration (y)**	**UPDRS-III**	**MMSE**	**MoCA**
			**PD**	**PDCI**	**PDD**	**HCs**	**DLB**	**PSP**	**AD**	**MSA-P**	**MSA-C**	**Mean** ±**SD**		**Mean** ±**SD**	**Mean** ±**SD**	**Mean** ±**SD**	**Mean** ±**SD**
Kepe et al. (20)	[^18^F] FDDNP	DVR	8	N/A	N/A	5	N/A	15	N/A	N/A	N/A	57.90 ± 10.00	50.00	2.40 ± 1.10	13.10 ± 6.70	28.50 ± 2.30	Un
Cho et al. (15)	[^18^F] AV-1451	SUVR	14	N/A	N/A	15	N/A	15	N/A	N/A	N/A	67.90 ± 5.40	53.33	5.50 ± 3.50	26.90 ± 11.20	26.90 ± 1.90	Un
Coakeley et al. (16)	[^18^F] AV-1451	SUVR	6	N/A	N/A	10	N/A	6	N/A	N/A	N/A	63.67 ± 9.61	50.00	5.50 ± 2.43	26.30 ± 3.01	Un	28.30 ± 0.72
Buongiorno et al. (8)	[^18^F] FDDNP	DVR	16	N/A	8	8	N/A	N/A	N/A	N/A	N/A	73.00 ± 4.25	60.00	Un	23.00 ± 8.00	29.00 ± 1.00	Un
Gomperts et al. (18)	[^18^F] AV-1451	SUVR	9	8	N/A	29	7	N/A	N/A	N/A	N/A	67.00 ± 3.00	88.89	Un	23.30 ± 12.80	29.10 ± 0.40	Un
Schonhaut et al. (25)	[^18^F] AV-1451	SUVR	26	N/A	N/A	46	N/A	33	N/A	N/A	N/A	67.10 ± 5.40	53.85	Un	26.10 ± 11.40	Un	Un
Hansen et al. (19)	[^18^F] AV-1451	SUVR	17	9	N/A	23	N/A	N/A	N/A	N/A	N/A	67.60 ± 6.30	76.47	4.50 ± 3.20	19.20 ± 8.40	28.90 ± 1.30	27.50 ± 1.70
Coakeley et al. (17)	[^18^F] AV-1451	SUVR	6	N/A	N/A	10	N/A	6	N/A	N/A	N/A	63.67 ± 9.61	50.00	5.50 ± 2.43	26.30 ± 3.01	Un	28.30 ± 0.72
Lee et al. (21)	[^18^F] AV-1451	SUVR	12	22	N/A	25	18	N/A	25	N/A	N/A	67.60 ± 6.30	58.33	3.51 ± 2.91	25.80 ± 11.30	27.20 ± 1.90	Un
Ossenkoppele et al. (27)	[^18^F] AV-1451	SUVR	23	70	N/A	160	24	40	83	N/A	N/A	67.30 ± 5.80	65.20	Un	Un	27.60 ± 1.90	Un
Schönecker et al. (24)	^18^F-THK-5351	SUVR	6	N/A	N/A	N/A	N/A	13	N/A	9	6	65.70 ± 8.10	33.33	Un	Un	29.40 ± 1.30	Un
Smith et al. (26)	[^18^F] AV-1451	SUVR	11	N/A	18	N/A	6	N/A	N/A	N/A	N/A	67.00 ± 5.50	72.73	Un	11.00 ± 8.00	28.00 ± 2.00	Un
Brendel et al. (14)	^18^F-PI-2620	SUVR	6	N/A	N/A	10	N/A	60	10	N/A	N/A	60.00 ± 10.00	66.67	1.42 ± 0.75	22.50 ± 6.30	Un	27.0 ± 4.0
Li et al. (22)	[^18^F] AV-1451	SUVR	8	N/A	N/A	10	N/A	7	4	N/A	N/A	66.40 ± 5.70	25.00	8.10 ± 6.60	18.10 ± 14.60	27.40 ± 4.30	Un
Li et al. (23)	^18^F-APN-1607	SUVR	10	N/A	N/A	13	N/A	20	N/A	7	N/A	59.40 ± 16.30	60.00	2.49 ± 1.44	38.90 ± 17.10	25.40 ± 4.30	Un

PD, Parkinson's disease; PDCI, Parkinson's disease with cognitive impairment; PDD, Parkinson's disease dementia; HCs, healthy controls; DLB, dementia with Lewy bodies; PSP, progressive supranuclear palsy; AD, Alzheimer's disease; MSA-P, multiple system atrophy–parkinsonian type; UPDRS, Unified Parkinson's Disease Rating Scale; MMSE, Mini-Mental State Examination; MoCA, Montreal Cognitive Assessment; SUVR, standard uptake value ratio; DVR, distribution volume ratios; SD, standard deviation.

### 3.3. Tau tracer uptake in PD patients compared with HCs

In total, 13 studies with 287 PD patients (90 PD [the cognitive status was not determined], 109 PDCI, 61 PDNC, and 27 PDND) and 363 HCs were included in this meta-analysis. We analyzed 30 brain regions (Table 2). Compared with HCs, PD patients had lower tau tracer binding in the substantia nigra (SMD = −0.81, 95% CI: [−1.32, −0.30], *I*^2^ = 62.2%) and hippocampus (SMD = −0.50, 95% CI: [−0.84, −0.16], *I*^2^ = 0). No difference in other brain regions was detected.

**Table 2:** Random effects meta-analyses results between PD and HCs subjects.

**Region**	**Number of studies**	**Subjects**		**SMD**	**[95% CI]**	**Overall effect**		**Heterogeneity**	
		**PD**	**HCs**			**Z**	**P**	**I** ^2^ **[%]**	***P***
Global	3	58	43	−0.102	[−0.69, 0.49]	−0.34	0.735	45.0	0.163
Frontal lobe	5	48	46	−0.39	[−1.07, 0.29]	−1.135	0.256	58.4	0.048
Prefrontal lobe	3	33	50	−0.14	[−0.59, 0.31]	−0.620	0.535	0	0.685
Parietal lobe	6	59	78	−0.08	[−0.58, 0.41]	−0.332	0.740	47.7	0.089
Sub.parietal lobe	2	49	40	0.128	[−0.29, 0.55]	0.60	0.551	0	0.442
Inf.parietal lobe	2	49	40	0.122	[−0.30, 0.54]	0.57	0.567	0	0.950
Occipital lobe	4	67	63	0.059	[−0.29, 0.41]	0.33	0.738	0	0.991
Lat.temporal lobe	3	34	26	0.12	[−0.40, 0.65]	0.459	0.646	0	0.405
Med.temporal lobe	2	24	13	−0.206	[−1.86, 1.45]	−0.240	0.807	79.9	0.026
Sup.temporal lobe	3	75	63	−0.18	[−0.52, 0.16]	−1.05	0.294	0	0.392
Mid.temporal lobe	2	49	40	0.056	[−0.364, 0.475]	0.26	0.795	0	0.481
Inf.temporal lobe	4	159	227	1.09	[−0.11, 2.28]	1.78	0.074	94.7	0
Striatum	2	18	18	0.10	[−0.73, 0.93]	0.234	0.815	31.9	0.226
Substantia nigra	6	89	117	−0.81	[−1.32,−0.30]	−3.10	**0.002**	62.2	0.021
Red nucleus	2	36	59	0.23	[−0.19, 0.65]	1.088	0.277	0	0.569
Caudate nucleus	4	57	84	−0.18	[−0.52, 0.16]	−1.034	0.301	0	0.469
Subthalamic nucleus	5	65	89	0.08	[−0.39, 0.55]	0.344	0.731	43.0	0.135
Putamen	6	62	99	0.03	[−0.29, 0.35]	0.169	0.866	0	0.420
Globus pallidus	7	97	127	−0.19	[−0.50, 0.12]	−1.21	0.228	19.5	0.281
Dentate nucleus	5	63	94	−0.00	[−0.32, 0.32]	−0.016	0.987	0	0.849
Thalamus	4	39	43	−0.11	[−0.55, 0.33]	−0.496	0.620	0	0.829
Midbrain	3	24	28	−0.01	[−0.57, 0.55]	−0.020	0.984	0	0.443
Ant.cingulate	2	49	40	−0.16	[−0.58, 0.26]	−0.76	0.448	0	0.648
Post.cingulate	4	73	53	−0.38	[−0.88, 0.12]	1.48	0.138	39.7	0.174
Entorhinal	4	168	223	−0.12	[−0.33, 0.08]	−1.18	0.237	0	0.852
Hippocampus	3	75	63	−0.50	[−0.84,−0.16]	−2.86	**0.004**	0	0.995
Precuneus	4	84	92	0.37	[−0.3, 1.05]	1.08	0.278	77.3	0.004
Sensorimotor	2	49	40	0.067	[−0.35, 0.49]	0.31	0.755	0	0.822
Insula	2	49	40	−0.15	[−0.57, 0.27]	0.71	0.477	0	0.888
Amygdala	2	49	40	−0.32	[−0.74, 0.104]	1.48	0.14	0	0.732

PD, Parkinson's disease; HCs, healthy controls; SMD, Standardized mean difference; CI, confidence interval. The bold values indicate the value of *P* < 0.05.

We performed subgroup analyses according to the types of tau tracers used (Table 3). We found that with first-generation tracers, PD patients had low tracer binding in the medial temporal lobe (SMD = −1.21, 95% CI: [−1.95, −0.47], *I*^2^ = 0), substantia nigra (SMD = −1.10, 95% CI: [−1.44, −0.75], *I*^2^ = 0), hippocampus (SMD = −0.50, 95% CI: [−0.84, −0.16], *I*^2^ = 0), and globus pallidus (SMD = −0.36, 95% CI: [−0.66, −0.06], *I*^2^ = 0). There was no difference in tau tracer binding between PD and HCs in the putamen, globus pallidus, subthalamic nucleus, midbrain, substantia nigra, and dentate nucleus.

**Table 3:** Subgroup analysis based on the type of tau tracer used for studies involving PD and HCs subjects.

**Region**	**Tracer**	**Number of studies**	**Subjects**		**SMD**	**[95% CI]**	**Overall effect**		**Heterogeneity**	
			**PD**	**HCs**			**Z**	**P**	**I** ^2^ **[%]**	***P***
Global	1st generation	3	58	43	−0.102	[−0.69, 0.49]	−0.34	0.735	45.0	0.163
	2nd generation	0	N/A	N/A	N/A	N/A	N/A	N/A	N/A	N/A
Frontal lobe	1st generation	4	38	33	−0.39	[−1.31, 0.52]	−0.847	0.397	68.7	0.022
	2nd generation	1	10	13	−0.37	[−1.20, 0.47]	−0.862	0.389	N/A	N/A
Prefrontal lobe	1st generation	2	27	40	−0.19	[−0.69, 0.30]	−0.760	0.447	0	0.461
	2nd generation	1	6	10	0.07	[−0.94, 1.09]	0.142	0.887	N/A	N/A
Parietal lobe	1st generation	5	49	65	−0.08	[−0.70, 0.54]	−0.253	0.801	58.1	0.049
	2nd generation	1	10	13	−0.10	[−0.93, 0.72]	−0.240	0.811	N/A	N/A
Sub.parietal lobe	1st generation	2	49	40	0.128	[−0.29, 0.55]	0.60	0.551	0	0.442
	2nd generation	0	N/A	N/A	N/A	N/A	N/A	N/A	N/A	N/A
Inf.parietal lobe	1st generation	2	49	40	0.122	[−0.30, 0.54]	0.57	0.567	0	0.950
	2nd generation	0	N/A	N/A	N/A	N/A	N/A	N/A	N/A	N/A
Occipital lobe	1st generation	3	57	50	0.072	[−0.31, 0.45]	−0.302	0.763	0	0.961
	2nd generation	1	10	13	0	[−0.82, 0.82]	0	1.000	N/A	N/A
Lat.temporal lobe	1st generation	2	24	13	0.35	[−0.33, 1.04]	1.013	0.311	0	0.391
	2nd generation	1	10	13	−0.21	[−1.04, 0.61]	−0.504	0.614		
Med.temporal lobe	1st generation	2	24	13	−1.21	[−1.95, −0.47]	−3.221	**0.001**	0	0.836
	2nd generation	0	N/A	N/A	N/A	N/A	N/A	N/A	N/A	N/A
Sup.temporal lobe	1st generation	3	75	63	−0.18	[−0.52, 0.16]	−1.05	0.294	0	0.392
	2nd generation	0	N/A	N/A	N/A	N/A	N/A	N/A	N/A	N/A
Mid.temporal lobe	1st generation	2	49	40	0.056	[−0.364, 0.475]	0.26	0.795	0	0.481
	2nd generation	0	N/A	N/A	N/A	N/A	N/A	N/A	N/A	N/A
Inf.temporal lobe	1st generation	4	159	227	1.09	[−0.11, 2.28]	1.78	0.074	94.7	0
	2nd generation	0	N/A	N/A	N/A	N/A	N/A	N/A	N/A	N/A
Caudate nucleus	1st generation	3	47	71	−0.20	[−0.62, 0.22]	−0.948	0.343	13.5	0.315
	2nd generation	1	10	13	0	[−0.82, 0.82]	0	1.000	N/A	N/A
Putamen	1st generation	4	46	76	−0.15	[−0.52, 0.22]	−0.791	0.429	0	0.725
	2nd generation	2	16	23	0.58	[−0.07, 1.24]	1.747	0.081	0	0.986
Globus pallidus	1st generation	5	81	104	−0.36	[−0.66, −0.06]	−2.38	**0.017**	0	0.699
	2nd generation	2	16	23	0.47	[−0.18, 1.12]	1.431	0.152	0	0.999
Thalamus	1st generation	3	29	30	−0.18	[−0.70, 0.34]	−0.675	0.500	0	0.721
	2nd generation	1	10	13	0.06	[−0.77, 0.88]	0.141	0.888	N/A	N/A
Subthalamic nucleus	1st generation	3	49	66	−0.09	[−0.85, 0.66]	−0.243	0.808	67.7	0.045
	2nd generation	2	16	23	0.31	[−0.33, 0.96]	0.950	0.342	0	0.488
Midbrain	1st generation	1	8	5	−0.62	[−1.77, 0.53]	−1.060	0.289	N/A	N/A
	2nd generation	2	16	23	0.19	[−0.45, 0.83]	0.570	0.568	0	0.671
Substantia nigra	1st generation	4	73	94	−1.18	[−1.52, −0.85]	−6.890	**0**	0	0.941
	2nd generation	2	16	23	0.13	[−0.51, 0.77]	0.396	0.692	0	0.624
Dentate nucleus	1st generation	3	47	71	0.05	[−0.32, 0.42]	0.254	0.799	0	0.626
	2nd generation	2	16	23	−0.15	[−0.79, 0.49]	−0.470	0.638	0	0.700
Entorhinal	1st generation	4	168	223	−0.12	[−0.33, 0.08]	−1.18	0.237	0	0.852
	2nd generation	0	N/A	N/A	N/A	N/A	N/A	N/A	N/A	N/A
Hippocampus	1st generation	3	75	63	−0.50	[−0.84,−0.16]	−2.86	**0.004**	0	0.995
	2nd generation	0	N/A	N/A	N/A	N/A	N/A	N/A	N/A	N/A
Precuneus	1st generation	4	84	92	0.37	[−0.3, 1.05]	1.08	0.278	77.3	0.004
	2nd generation	0	N/A	N/A	N/A	N/A	N/A	N/A	N/A	N/A
Post.cingulate	1st generation	4	73	53	−0.38	[−0.88, 0.12]	1.48	0.138	39.7	0.174
	2nd generation	0	N/A	N/A	N/A	N/A	N/A	N/A	N/A	N/A
Ant.cingulate	1st generation	2	49	40	−0.16	[−0.58, 0.26]	−0.76	0.448	0	0.648
	2nd generation	0	N/A	N/A	N/A	N/A	N/A	N/A	N/A	N/A
Sensorimotor	1st generation	2	49	40	0.067	[−0.35, 0.49]	0.31	0.755	0	0.822
	2nd generation	0	N/A	N/A	N/A	N/A	N/A	N/A	N/A	N/A
Insula	1st generation	2	49	40	−0.15	[−0.57, 0.27]	0.71	0.477	0	0.888
	2nd generation	0	N/A	N/A	N/A	N/A	N/A	N/A	N/A	N/A
Amygdala	1st generation	2	49	40	−0.32	[−0.74, 0.10]	1.48	0.14	0	0.732
	2nd generation	0	N/A	N/A	N/A	N/A	N/A	N/A	N/A	N/A

PD, Parkinson's disease; HCs, healthy controls; SMD, Standardized mean difference; CI, confidence interval; N/A, Not applicable. The bold values indicate the value of *P* < 0.05.

Publication bias revealed by Egger's test was not significant, and sensitivity analysis showed no difference in results (Supplementary Table 3).

### 3.4. Tau tracer uptake in PDCI patients compared with HCs

A total of four studies with 109 PDCI patients and 237 HCs were included. The 109 PDCI patients included 96 PD-MCI patients and 13 PDD patients, but the tau tracer uptake values of these 13 PDD patients were not separated from those of PD-MCI patients. The tracer used in all four studies was ^18^F-AV-1451. The results showed that PDCI patients had higher tau uptake values in the inferior temporal lobe (SMD = 1.583, 95% CI: [0.049, 3.116], *I*^2^ = 94.8%) than HCs. PDCI patients and HCs showed no difference in tau tracer binding in the entorhinal, hippocampus, superior temporal lobe, middle and inferior temporal lobe, and precuneus (Table 4). Publication bias revealed by Egger's test was not significant, and sensitivity analysis showed no difference in results (Supplementary Table 4).

**Table 4:** Random effects meta-analyses results between PDCI and HCs subjects.

**Region**	**Number of studies**	**Subjects**		**SMD**	**[95% CI]**	**Overall effect**		**Heterogeneity**	
		**PDCl**	**HCs**			**Z**	**P**	**I** ^2^ **[%]**	***P***
Entorhinal	3	101	208	0.059	[−0.181, 0.299]	0.48	0.630	0	0.934
Hippocampus	2	31	48	−0.357	[−0.821, 0.107]	1.51	0.132	0	0.549
Sup.temporal lobe	2	31	48	0.033	[−0.458, 0.524]	0.13	0.894	10.1	0.292
Mid-inf.temporal lobe	2	31	48	0.275	[−0.188, 0.737]	1.16	0.244	0	0.581
Inf.temporal lobe	3	100	214	1.583	[0.049, 3.116]	2.02	**0.043**	94.8	0
Precuneus	3	39	77	1.838	[−0.413, 4.089]	1.60	0.109	95.21	0

PDCI, Parkinson's disease with cognitive impairment; HCs, healthy controls; SMD, Standardized mean difference; CI, confidence interval. The bold values indicate the value of *P* < 0.05.

### 3.5. Tau tracer uptake in PDCI patients compared with PDNC patients

A total of 109 patients with PDCI (96 PD-MCI, 13 PDD) and 61 patients with PDNC were reported in four studies, and ^18^F-AV-1451 was used as the tracer in all of these studies. The mean age of PDNC was 67 years, and the age of PDCI ranged from 69 to 72 years. The tracer binding of PDCI in the entorhinal region (SMD = 0.55, 95% CI: [0.19, 0.91], *I*^2^ = 0) was higher than that in PDNC, and there was no difference in tracer binding between the two groups in the hippocampus, superior temporal lobe, middle and inferior temporal lobe, inferior temporal lobe, and precuneus (Table 5). Publication bias revealed by Egger's test was not significant, and sensitivity analysis showed no difference in results (Supplementary Table 5).

**Table 5:** Random effects meta-analyses results between PDCI and PDNC subjects.

**Region**	**Number of studies**	**Subjects**		**SMD**	**[95% CI]**	**Overall effect**		**Heterogeneity**	
		**PDCI**	**PDNC**			**Z**	**P**	**I** ^2^ **[%]**	***P***
**PDCI vs. PDNC**									
Entorhinal	3	101	52	0.55	[0.19, 0.91]	−3.000	**0.003**	0	0.785
Hippocampus	2	31	29	0.19	[−0.76, 0.39]	−0.641	0.522	12.7	0.285
Sup.temporal lobe	2	31	29	0.27	[−0.80, 0.27]	−0.982	0.326	0	0.603
Mid-inf.temporal lobe	2	31	29	0.50	[−1.04, 0.04]	−1.815	0.070	0	0.777
Inf.temporal lobe	3	100	44	1.17	[−2.37, 0.03]	−1.910	0.056	85.2	0.001
Precuneus	3	39	38	0.98	[−2.16, 0.20]	−1.634	0.102	79.7	0.007

PDCI, Parkinson's disease with cognitive impairment; PDNC, Parkinson's disease with normal cognition; SMD, Standardized mean difference; CI, confidence interval. The bold values indicate the value of *P* < 0.05.

### 3.6. Tau tracer uptake in PDD patients compared with PDND patients

In total, two studies reported 26 PDD patients and 27 PDND patients using tracers ^18^F-AV-1451 and ^18^F-FDDNP were included in our studies; one (8) study showed that the uptake of tau tracer in the lateral temporal lobe region of PDD patients was higher than that of PDND. But another (26) study showed that there was no difference in the uptake value of the two groups in the temporal lobe, and PDD patients had higher uptake values in the medial parietal lobe and lower uptake values in the substantia nigra. There were some differences in the age of the subjects in the two studies. The mean age of PDND in Smith's study was 67 years old, and the age of PDD was 73 years old. In another study, the age of PDND was 73, and the age of PDD was 78. The UPDRS-III score of the subjects in the two groups was also significantly different. In Smith's (26) study, the score of PDND was 11, and the score of PDD was 27; in Buongiorno et al.'s (8) study, the PDND score was 23 and the PDD score was 34.5.

### 3.7. Tau tracer uptake in PD patients compared with PSP patients

There were 10 studies including 113 PD (90 PD [the cognitive status was not determined], 23 PDNC) patients and 215 PSP patients. We analyzed 21 brain regions (Table 6). Compared to PSP, PD patients showed a reduction of tracer binding in the midbrain, subthalamic nucleus, globus pallidus, cerebellar deep white matter, thalamus, striatum, substantia nigra, dentate nucleus, red nucleus, putamen, and frontal lobe (SMD range: −2.16 to −0.55, I^2^ range: 0 to 74.3, specific data are listed in Table 6), in order of decreasing SMD absolute value.

**Table 6:** Random effects meta-analyses results between PD and PSP subjects.

**Region**	**Number of studies**	**Subjects**		**SMD**	**[95% CI]**	**Overall effect**		**Heterogeneity**	
		**PD**	**HCs**			**Z**	**P**	**I** ^2^ **[%]**	***P***
Frontal lobe	4	32	48	−0.55	[−1.06, −0.04]	−2.124	**0.034**	13.4	0.325
Prefrontal lobe	2	21	74	0.06	[−0.49, 0.61]	0.204	0.838	0	0.814
Parietal lobe	5	47	62	−0.30	[−0.84, 0.23]	−1.113	0.266	42.6	0.138
Occipital lobe	3	33	41	−0.05	[−0.58, 0.48]	−0.192	0.848	20.2	0.286
Temporal lobe	3	33	49	−0.32	[−0.78, 0.13]	−1.394	0.163	0	0.508
Lat.temporal lobe	2	16	22	−0.65	[−1.33, 0.02]	−1.891	0.059	0	0.706
Striatum	3	24	53	−1.28	[−2.21, −0.34]	−2.659	**0.008**	64.9	0.058
Caudate nucleus	4	57	73	0.30	[−0.17, 0.77]	1.257	0.209	36.1	0.195
Putamen	6	71	140	−0.86	[−1.33, −0.40]	−3.636	**0**	46.0	0.099
Globus pallidus	6	71	140	−1.77	[−2.53, −1.02]	−4.614	**0**	73.3	0.002
Thalamus	4	39	55	−1.44	[−2.42, −0.47]	−2.904	**0.004**	74.3	0.009
Subthalamic nucleus	5	65	142	−2.05	[−2.77, −1.32]	−5.532	**0**	68.8	0.012
Midbrain	3	24	48	−2.16	[−3.22, −1.10]	−4.007	**0**	63.6	0.064
Substantia nigra	5	63	133	−1.12	[−1.59, −0.65]	−4.640	**0**	40.8	0.149
Red nucleus	2	36	53	−0.90	[−1.68, −0.12]	−2.256	**0.024**	61.1	0.109
Pons	2	32	46	−0.67	[−1.99, 0.65]	−0.996	0.319	79.7	0.026
Dentate nucleus	5	63	133	−1.00	[−1.58, −0.42]	−3.358	**0.001**	62.2	0.032
Cerebellar deep white matter	2	14	28	−1.77	[−3.13, −0.41]	−2.557	**0.011**	67.8	0.078
Post.cingulate	2	23	29	−0.92	[−2.80, 0.96]	−0.962	0.336	88.7	0.003
Entorhinal	2	28	54	−0.24	[−0.66, 0.18]	−1.123	0.261	0	0.952
Inf.temporal lobe	2	28	54	−0.35	[−0.77, 0.08]	−1.609	0.108	0	0.500

PD, Parkinson's disease; PSP, progressive supranuclear palsy; SMD, Standardized mean difference; CI, confidence interval. The bold values indicate the value of *P* < 0.05.

Subgroup analysis was performed according to the type of tracer (Table 7). In the subgroup using first-generation tracers (^18^F-AV-1451 [*n* = 6], ^18^F-FDDNP [*n* = 1], and ^18^F-THK-5351 [*n* = 1]), a total of 18 brain regions were compared. Compared to PSP, PD patients had lower binding to tau tracers in the following regions: midbrain, subthalamic nucleus, globus pallidus, cerebellar deep white matter, thalamus, striatum, substantia nigra, and dentate nucleus (SMD range: −2.68 to −0.82, *I*^2^ range: 0 to 81.6, specific data are listed in Table 7). In the subgroup using second-generation tracers (^18^F-PI-2620 [*n* = 1] and ^18^F-APN-1607 [*n* = 1]), a total of five brain regions were compared, including the putamen, globus pallidus, subthalamic nucleus, substantia nigra, and dentate nucleus. PD patients had low tau tracers binding in the globus pallidus, substantia nigra, subthalamic nucleus, dentate nucleus, and putamen (SMD range: −1.41 to −1.09, *I*^2^ range: 0 to 76.4, specific data are listed in Table 7). Publication bias revealed by Egger's test was not significant, and sensitivity analysis showed no difference in results (Supplementary Table 6).

**Table 7:** Subgroup analysis based on the type of tau tracer used for studies involving PSP and PD subjects.

**Region**	**Tracer**	**Number of studies**	**Subjects**		**SMD**	**[95% CI]**	**Overall effect**		**Heterogeneity**	
			**PD**	**PSP**			**Z**	***P***	**I** ^2^ **[%]**	***P***
Frontal lobe	1st generation	3	22	28	−0.62	[−1.40, 0.16]	−1.561	0.119	40.1	0.188
	2nd generation	1	10	20	−0.44	[−1.21, 0.33]	−1.118	0.264	N/A	N/A
Parietal lobe	1st generation	4	37	42	−0.31	[−1.04, 0.41]	−0.839	0.401	56.8	0.074
	2nd generation	1	10	20	−0.32	[−1.08, 0.45]	−0.809	0.418	N/A	N/A
Occipital lobe	1st generation	2	23	21	0.03	[−0.83, 0.90]	0.077	0.939	48.3	0.164
	2nd generation	1	10	20	−0.27	[−1.03, 0.49]	−0.693	0.488	N/A	N/A
Temporal lobe	1st generation	2	23	29	−0.27	[−0.90, 0.35]	−0.858	0.391	18.6	0.268
	2nd generation	1	10	20	−0.43	[−1.20, 0.33]	−1.107	0.268	N/A	N/A
Striatum	1st generation	2	14	28	−1.69	[−2.71, −0.67]	−3.238	**0.001**	45.5	0.175
	2nd generation	1	10	20	−0.60	[−1.37, 0.18]	−1.507	0.132	N/A	N/A
Caudate nucleus	1st generation	3	47	53	0.39	[−0.21, 0.98]	1.276	0.202	45.6	0.159
	2nd generation	1	10	20	0.00	[−0.76, 0.76]	0	1.000	N/A	N/A
Putamen	1st generation	4	55	60	−0.70	[−1.40, 0.00]	−1.954	0.051	62.7	0.045
	2nd generation	2	16	80	−1.09	[−1.68, −0.50]	−3.643	**0**	0	0.419
Globus pallidus	1st generation	4	55	60	−1.98	[−3.18, −0.78]	−3.241	**0.001**	81.6	0.001
	2nd generation	2	16	80	−1.41	[−2.02, −0.80]	−4.520	**0**	0	0.640
Thalamus	1st generation	3	29	35	−1.71	[−3.14, −0.28]	−2.348	**0.019**	80.5	0.006
	2nd generation	1	10	20	−0.85	[−1.64, −0.06]	−2.099	**0.036**	N/A	N/A
Subthalamic nucleus	1st generation	3	49	62	−2.58	[−3.71, −1.45]	−4.484	**0**	74.6	0.019
	2nd generation	2	16	80	−1.37	[−1.98, −0.76]	−4.384	**0**	0	0.378
Midbrain	1st generation	2	14	28	−2.68	[−3.56, −1.79]	−5.920	**0**	0	0.325
	2nd generation	1	10	20	−1.35	[−2.19, −0.52]	−3.167	**0.002**	N/A	N/A
Substantia nigra	1st generation	3	47	53	−0.94	[−1.68, −0.20]	−2.497	**0.013**	59.3	0.086
	2nd generation	2	16	80	−1.41	[−2.02, −0.80]	−4.566	**0**	0	0.783
Pons	1st generation	2	32	46	−0.67	[−1.99, 0.65]	−0.996	0.319	79.7	0.026
	2nd generation	0	N/A	N/A	N/A	N/A	N/A	N/A	N/A	N/A
Dentate nucleus	1st generation	3	47	53	−0.82	[−1.62, −0.02]	−2.000	**0.046**	67.0	0.048
	2nd generation	2	16	80	−1.28	[−2.51, −0.05]	−2.043	**0.041**	76.4	0.040
Cerebellar deep white matter	1st generation	2	14	28	−1.77	[−3.13, −0.41]	−2.557	**0.011**	67.8	0.078
Post.cingulate	1st generation	2	23	29	−0.92	[−2.80, 0.96]	−0.962	0.336	88.7	0.003
	2nd generation	0	N/A	N/A	N/A	N/A	N/A	N/A	N/A	N/A
Entorhinal	1st generation	2	28	54	−0.24	[−0.66, 0.18]	−1.123	0.261	0	0.952
	2nd generation	0	N/A	N/A	N/A	N/A	N/A	N/A	N/A	N/A
Inf.temporal lobe	1st generation	2	28	54	−0.35	[−0.77, 0.08]	−1.609	0.108	0	0.500
	2nd generation	0	N/A	N/A	N/A	N/A	N/A	N/A	N/A	N/A

PD, Parkinson's disease; PSP, progressive supranuclear palsy; SMD, Standardized mean difference; CI, confidence interval; N/A, Not applicable. The bold values indicate the value of *P* < 0.05.

### 3.8. Tau tracer uptake in PD patients compared with AD patients

A total of 49 PD patients (14 PD [the cognitive status was not determined], 35 PDNC) and 122 AD patients from four studies were included in the meta-analysis. The average age of PD patients in Ossenkoppele's (27) study was 60 years old, while the average age of PD patients in the other three studies was 67 years old. The age of AD patients ranged from 70 to 74 years old. Three studies used ^18^F-AV-1451, and one study used ^18^F-PI-2620. The results showed that PD patients had lower tracer binding in the frontal lobe (SMD = −2.21, 95% CI: [−4.11, −0.31], *I*^2^ = 83.9%) and occipital lobe (SMD = −1.70, 95% CI: [−2.62, −0.78], *I*^2^ = 62.0%). There was no difference in tau tracer binding in temporal and occipital lobes between PD patients and AD patients (Table 8). Publication bias revealed by Egger's test was not significant, and sensitivity analysis showed no difference in results (Supplementary Table 7).

**Table 8:** Random effects meta-analyses results between PD and AD subjects.

**Region**	**Number of studies**	**Subjects**		**SMD**	**[95% CI]**	**Overall effect**		**Heterogeneity**	
		**PD**	**AD**			**Z**	**P**	**I** ^2^ **[%]**	***P***
Global	2	20	29	−3.27	[−6.86, 0.32]	−1.785	0.074	85.6	0.008
Frontal lobe	3	26	39	−2.21	[−4.11, −0.31]	−2.274	**0.023**	83.9	0.002
Parietal lobe	2	20	29	−2.75	[−6.15, 0.65]	−1.585	0.113	86.7	0.006
Occipital lobe	2	20	29	−1.70	[−2.62, −0.78]	−3.625	**0**	26.0	0.245
Temporal lobe	2	20	29	−2.75	[−5.75, 0.24]	−1.804	0.071	83.8	0.013

PD, Parkinson's disease; AD, Alzheimer's disease; SMD, Standardized mean difference; CI, confidence interval. The bold values indicate the value of *P* < 0.05.

### 3.9. Tau tracer uptake in PD patients compared with DLB patients

A total of four studies were included in our meta-analysis, including 55 PD patients (11 PD [the cognitive status was not determined], 44 PDNC) and 55 DLB patients. There was no difference in the age of PD patients. DLB patients also showed little age difference, ranging from 68 to 73 years old. The tracer used in the four studies is ^18^F-AV-1451. The brain regions included the frontal lobe, parietal lobe, occipital lobe, and inferior temporal lobe (Table 9). Compared with DLB, PD patients had lower tracer binding in the inferior temporal lobe (SMD = −1.49, 95% CI: [−2.34, −0.63], *I*^2^ = 69.6%) and occipital lobe (SMD = −0.74, 95% CI: [−1.35, −0.13], *I*^2^ = 0). Publication bias revealed by Egger's test was not significant, and sensitivity analysis showed no difference in results (Supplementary Table 8).

**Table 9:** Random effects meta-analyses result between PD and DLB subjects.

**Region**	**Number of studies**	**Subjects**		**SMD**	**[95% CI]**	**Overall effect**		**Heterogeneity**	
		**PD**	**DLB**			**Z**	**P**	**I** ^2^ **[%]**	***P***
Frontal lobe	2	23	24	−0.47	[−1.25, 0.31]	−1.181	0.238	37.2	0.207
Occipital lobe	2	23	24	−0.74	[−1.35, −0.13]	−2.375	**0.018**	0	0.580
Parietal lobe	2	23	24	−1.13	[−2.28, 0.02]	−1.918	0.055	63.8	0.096
Inf.temporal lobe	4	55	55	−1.49	[−2.34, −0.63]	−3.392	**0.001**	69.6	0.02

PD, Parkinson's disease; DLB, dementia with Lewy bodies; SMD, Standardized mean difference; CI, confidence interval. The bold values indicate the value of *P* < 0.05.

### 3.10. Tau tracer uptake in PD patients compared with MSA patients

In total, two studies reported 16 PD (the cognitive status was not determined) patients and 16 MSA-P patients were included in our study. Since the brain regions analyzed in the two articles were not consistent, we did not perform a meta-analysis. The results of one study (23) confirmed that the ^18^F-APN-1607 was bound in the putamen of MSA-P patients (4 of 7) but not in PD patients, and in another study (24), it was found that PD patients had lower ^18^F-THK-5351 uptake in the lentiform nucleus than MSA-P patients. This study (24) also compared tracer uptake between MSA-C patients and PD patients and showed that MSA-C patients had significantly higher ^18^F-THK-5351 uptake in the pons. It was also elevated when compared to MSA-P but did not survive the significance threshold for multiple comparisons.

## 4. Discussion

To the best of our knowledge, this is the first comprehensive systematic review and meta-analysis of tau PET imaging in PD. The results showed that PDCI patients had higher tau tracer uptake in the inferior temporal lobe than HCs and had higher tau tracer uptake in the entorhinal region than PDNC patients. Tau tracer uptake values in PD patients were lower than PSP patients in the midbrain, subthalamic nucleus, globus pallidus, cerebellar deep white matter, thalamus, striatum, substantia nigra, dentate nucleus, red nucleus, putamen, and frontal lobe and lower than those in patients with AD in the frontal lobe and occipital lobe.

There is heterogeneity in tau protein burden in PD patients. Pathological studies have shown that the features of PDCI are subcortical PD changes and cortical changes consistent with AD (28–31), namely, the presence of not only α-Syn-rich Lewy bodies but also the deposition of β-amyloid and hyperphosphorylated 3R/4R tau in the cerebral cortex in PD patients. Conversely, coexisting AD pathology in PD patients in the absence of cognitive impairment is rare (28, 30, 32). The results of our meta-analysis show that the anatomic localization for abnormal ^18^F-AV-1451 binding in PDCI patients is similar to that reported in individuals with AD (33–36), including the inferior temporal and entorhinal cortical regions. The uptake value of the tau tracer in the inferior temporal lobe of PDCI patients was higher than that of HCs, and the uptake value of the tau tracer in the entorhinal region of PDCI patients was higher than that of PDNC patients. This finding may suggest that the neuropathologic processes that drive the accumulation of tau deposits in PDCI patients have the same regional vulnerabilities as those driving tau deposition in individuals with AD patients.

The different tau tracers play a significant impact on the results of tau PET studies measuring tau burden. First-generation tracers used in the studies included in the meta-analysis were ^18^F-FDDNP and ^18^F-AV-1451. One study used the ^18^F-FDDNP tracer, which has limited clinical application due to its lack of specificity and selectivity for *in vivo* imaging and it is binding to neurofibrillary tangles and amyloid plaques in the brains. ^18^F-AV-1451 is the most widely used tracer in patients with neurodegenerative diseases, and a recent study (37, 38) with postmortem tissue has confirmed that ^18^F-AV-1451 binds strongly to tau in neurofibrillary tangles and neurites without binding Aβ and has shown, critically, that ^18^F-AV-1451 does not bind α-Syn aggregates or Lewy bodies. In this meta-analysis, ^18^F-AV-1451 could find tau lesions in patients with PDCI. However, it has also been shown to off-target neuromelanin in the substantia nigra (37). Our meta-analysis found that the binding of ^18^F-AV-1451 in patients with PD was significantly reduced in the substantia nigra compared to HCs. This can be explained by the fact that ^18^F-AV-1451 can bind to neuromelanin in normal neurons, while PD patients have decreased neuromelanin in the substantia nigra.

Currently, second-generation tau tracers have also been used in this field, optimizing the shortcomings of off-target binding of the first-generation tracers (39). There were two studies using second-generation tracers in PD patients, using ^18^F-PI-2620 and ^18^F-APN-1607, respectively. ^18^F-APN-1607 showed a higher signal-to-background ratio and less off-target signal in the basal ganglia than the first-generation tracer (39). Another second-generation tracer ^18^F-PI-2620 also showed a lack of non-target binding in areas recorded in the choroid plexus, basal ganglia, striatum, amygdala, meninges, or other first-generation tau reagents (40). Our meta-analysis showed no difference in tau tracer uptake values between PD and HCs when using second-generation tracers. Overall, the results suggest that second-generation tau tracers show great promise as PDCI biomarkers.

Tau pathology is now considered a key pathogenic mechanism of various neurodegenerative diseases, and its characteristics vary according to the brain regions affected by tau aggregation. Our meta-analysis found that PD patients had lower tau uptake values in the midbrain, subthalamic nucleus, globus pallidus, cerebellar deep white matter, thalamus, striatum, substantia nigra, dentate nucleus, red nucleus, putamen, and frontal lobe than PSP patients. This was consistent with the findings of postmortem brain studies of PSP patients, where neuronal tau aggregates first appeared in the globus pallidus, subthalamic nucleus, and substantia nigra, followed by the midbrain, and then accumulated in the striatum, dentate nucleus, frontal lobe are involved at a later stage (41, 42). No differences were found in the parietal lobe, temporal lobe, or occipital lobe; probably due to the relatively short duration of PSP patients in our included study, these cortical regions had not been affected by tau pathology. However, it should be noted that the type of tau deposition in PD was mainly AD-type, characterized by hyperphosphorylated 3R/4R tau deposition, while PSP was a tau lesion associated with the 4R tau subtype. In various neurodegenerative diseases, the strength of ^18^F-AV-1451 binding with respect to tau isoforms in various neurodegenerative disorders was 3R + 4R tau > 3R tau (e.g., Pick disease) or 4R tau (43). Preliminary *in vitro* experiments with second-generation radiotracers have shown that not only they were highly selectively bound to Alzheimer's type NFTs but also 3R and 4R-rich brain homogenated from patients with Pick disease and PSP (14, 44–46).

The results of the meta-analysis showed that AD patients had higher tau uptake values in the frontal and occipital lobes than in PD. However, no differences were found in the temporal lobe, where tau pathology accumulated earlier in AD patients (47). This may be due to the fact that the PD patients we included were not classified according to their cognitive status, and some patients had AD-like tau pathology. Even if cortical tau tracer binding was elevated in PDCI participants, it was much lower than in AD patients, consistent with neuropathologic reports (48).

DLB had increased tau uptake in the occipital lobe and inferior temporal lobe compared to PD. This is consistent with previously reported autopsy findings that clinically diagnosed patients with suspected DLB had various pathological burdens of hyperphosphorylated tau and α-Syn, with the greatest burden of hyperphosphorylated tau occurring in the occipital lobe, followed by the temporal lobe (49). Due to the small sample size, our conclusions need more research to support the future.

Multiple system atrophy—parkinsonian type belongs to the same α-Syn disease as PD. Although it is not a tauopathy, MSA-P patients had a higher lentiform nucleus tau tracer uptake value than PD patients. This result can be explained by the off-target binding of the tau tracer. ^18^F-THK-5351 and ^18^F-APN-1607 were used in the two studies comparing the tau burden of MSA-P and PD patients. ^18^F-THK5351 has been shown to bind off-target to monoamine oxidase-B (MAO-B) (50–52), and an autopsy study showed that MAO-B levels were elevated in the putamen of MSA patients but not in the basal ganglia of PD patients (53). MAO-B is also elevated in the pontine region of MSA-C patients because this area is an early focus of α-Syn (54), and other studies have confirmed that elevated MAO-B levels in MSA are associated with the presence of oligodendrocytes containing α-Syn glial cytoplasmic inclusions (55). This also explains the detection of large tracer uptake in the pontine region in the MSA-C patient group.

This study has certain limitations. First, only four of the 13 studies classified PD into PDNC and PDCI, and most of the studies did not classify PD according to the stage of cognitive impairment. Second, we lack data on patients with PD-MCI, and the sample size of PDD patients is relatively small. This prevented us from performing a meta-analysis of tau deposition in PD patients at various stages of cognitive impairment. Third, all PDCI patients were treated with the first-generation tracer ^18^F-AV-1451, and no study was performed using the second-generation tracer. Fourth, different PET-CT scanners, tracers, and participant characteristics increased the risk of heterogeneity. However, due to the limited number of studies, subgroup analysis and meta-regression could not be performed to explore the source of heterogeneity. Moreover, cognitive impairment in PDCI patients can be caused not only by pathological deposition of tau protein in the cerebral cortex but also by cerebrovascular factors or α-Syn diffusion in the brain.

In conclusion, advances in *in vivo* tau imaging have provided exciting and promising results for the use of tau PET in dementia research. This meta-analysis shows that tau PET may be helpful for *in vivo* visualization and quantitation of tau pathology in PDCI. The region-specific binding pattern of tau tracers may be helpful in the differential diagnosis between Parkinson's disease and other neurodegenerative diseases. We look forward to more tau PET studies in PD patients in the future, preferably with large sample sizes, to explore the longitudinal distribution characteristics of tau protein in PD patients at different cognitive stages (PDNC, PD-MCI, and PDD) in more detail. Since first-generation tau PET tracers mainly bind to the typical 3R/4R tau deposition of AD and exhibit off-target binding, second-generation tau PET tracers with higher binding affinity and selectivity were developed. Second-generation tracers have less off-target binding than first-generation tracers and can bind all types of tau deposition with high selectivity. All these results demonstrate the potential of second-generation tracers. However, a more in-depth study is still needed in the future. Several novel tau tracers are currently progressing in clinical human studies, and we eagerly await the results.

## Data availability statement

The original contributions presented in the study are included in the article/Supplementary material, further inquiries can be directed to the corresponding authors.

## Author contributions

JZ, JJ, DS, and TF: conception and organization of the study. JZ, JJ, and TF: execution of the search and article selection. JZ, DS, and TF: data extraction and statistical analysis. JZ: writing of the first draft. TF, JJ, and DS: review and critique of the manuscript. All authors contributed to the article and approved the submitted version.
