# Supplementary material for: Tau-PET imaging in Parkinson's disease: a systematic review and meta-analysis
Source: Front Neurol. 2023 Apr 27;14:1145939. doi: 10.3389/fneur.2023.1145939 (PMC10174250; doi:10.3389/fneur.2023.1145939)
Supplement: Supplementary file 1 [file Data_Sheet_1.ZIP › Supplementary/Supplementary Table 1.docx]

**Supplemental Table 1. Search strategy in Pubmed/MEDLINE, EmBase, Cochrane Library and Web of Science.**

*Date of last search: June 1, 2022.*

| **Database** | **Search** |
| --- | --- |
| **Pubmed / MEDLINE** | ((((((((((((((Parkinson Disease[Title/Abstract]) OR (Idiopathic Parkinson's Disease[Title/Abstract])) OR (Lewy Body Parkinson's Disease[Title/Abstract])) OR (Parkinson's Disease, Idiopathic[Title/Abstract])) OR (Parkinson's Disease, Lewy Body[Title/Abstract])) OR (Parkinson Disease, Idiopathic[Title/Abstract])) OR (Parkinson's Disease[Title/Abstract])) OR (Idiopathic Parkinson Disease[Title/Abstract])) OR (Lewy Body Parkinson Disease[Title/Abstract])) OR (Primary Parkinsonism[Title/Abstract])) OR (Parkinsonism, Primary[Title/Abstract])) OR (Paralysis Agitans[Title/Abstract])) OR (PD[Title/Abstract])) OR (((((((Diffuse Lewy Body Disease[Title/Abstract]) OR (Lewy Body Dementia[Title/Abstract])) OR (Cortical Lewy Body Disease[Title/Abstract])) OR (Lewy Body Disease, Cortical[Title/Abstract])) OR (Lewy Body Type Senile Dementia[Title/Abstract])) OR (Lewy Body Disease, Diffuse[Title/Abstract])) OR (Dementia, Lewy Body[Title/Abstract])) OR ("Lewy Body Disease"[Mesh]) AND (((((((tau Proteins[Title/Abstract]) OR (Proteins, tau[Title/Abstract])) OR (tau Protein[Title/Abstract])) OR (Protein, tau[Title/Abstract])) OR (tau[Title/Abstract])) OR (Tauopathies[Title/Abstract])) OR (Tauopathy[Title/Abstract]))) AND ((((((((((((((((((((((((((((((((((Positron-Emission Tomography[Title/Abstract]) OR (Positron Emission Tomography[Title/Abstract])) OR (Positron-Emission Tomography Imaging[Title/Abstract])) OR (Imaging, Positron-Emission Tomography[Title/Abstract])) OR (Positron Emission Tomography Imaging[Title/Abstract])) OR (Positron-Emission Tomography Imagings[Title/Abstract])) OR (Tomography Imaging, Positron-Emission[Title/Abstract])) OR (Tomography, Positron-Emission[Title/Abstract])) OR (Tomography, Positron Emission[Title/Abstract])) OR (PET Scan[Title/Abstract])) OR (PET Scans[Title/Abstract])) OR (Scan, PET[Title/Abstract])) OR (PET Imaging[Title/Abstract])) OR (Imaging, PET[Title/Abstract])) OR (PET Imagings[Title/Abstract])) OR (Tomography, Emission-Computed, Single-Photon[Title/Abstract])) OR (CT Scan, Single-Photon Emission[Title/Abstract])) OR (CT Scan, Single Photon Emission[Title/Abstract])) OR (Radionuclide Tomography, Single-Photon Emission-Computed[Title/Abstract])) OR (Radionuclide Tomography, Single Photon Emission Computed[Title/Abstract])) OR (Tomography, Single-Photon, Emission-Computed[Title/Abstract])) OR (Single-Photon Emission Computerized Tomography[Title/Abstract])) OR (Single Photon Emission Computerized Tomography[Title/Abstract])) OR (Single-Photon Emission CT Scan[Title/Abstract])) OR (Single Photon Emission CT Scan[Title/Abstract])) OR (Single-Photon Emission-Computed Tomography[Title/Abstract])) OR (Emission-Computed Tomography, Single-Photon[Title/Abstract])) OR (Single Photon Emission Computed Tomography[Title/Abstract])) OR (Tomography, Single-Photon Emission-Computed[Title/Abstract])) OR (SPECT[Title/Abstract])) OR (CAT Scan, Single-Photon Emission[Title/Abstract])) OR (CAT Scan, Single Photon Emission[Title/Abstract])) OR (Single-Photon Emission Computer-Assisted Tomography[Title/Abstract])) OR (Single Photon Emission Computer Assisted Tomography[Title/Abstract])) |
| **Cochrane Library** | MeSH descriptor: [Parkinson Disease] explode all trees OR (Idiopathic Parkinson's Disease):ti,ab,kw OR (Lewy Body Parkinson's Disease):ti,ab,kw OR (Parkinson's Disease, Idiopathic):ti,ab,kw OR (Parkinson's Disease, Lewy Body):ti,ab,kw OR (Parkinson Disease, Idiopathic):ti,ab,kw (Word variations have been searched) OR (Parkinson's Disease):ti,ab,kw OR (Idiopathic Parkinson Disease):ti,ab,kw OR (Lewy Body Parkinson Disease):ti,ab,kw OR (Primary Parkinsonism):ti,ab,kw OR (Parkinsonism, Primary):ti,ab,kw (Word variations have been searched) OR (Paralysis Agitans):ti,ab,kw OR (lewy body disease):ti,ab,kw OR (dementia, lewy body):ti,ab,kw OR (lewy body disease, diffuse):ti,ab,kw OR (lewy body type senile dementi):ti,ab,kw OR (lewy body disease, cortical):ti,ab,kw OR (cortical lewy body disease):ti,ab,kw OR (lewy body dementia):ti,ab,kw OR (diffuse lewy body disease):ti,ab,kw AND (tauopathies):ti,ab,kw OR (tauopathy):ti,ab,kw OR (tau proteins):ti,ab,kw OR (proteins, tau):ti,ab,kw OR (tau protein):ti,ab,kw OR (protein, tau):ti,ab,kw OR (tau):ti,ab,kw AND (tomography, single-photon emission-computed):ti,ab,kw OR (spect):ti,ab,kw OR (cat scan, single-photon emission):ti,ab,kw OR (cat scan, single photon emission):ti,ab,kw OR (single-photon emission computer-assisted tomography):ti,ab,kw OR (single photon emission computer assisted tomography):ti,ab,kw OR (single-photon emission computerized tomography):ti,ab,kw OR (single photon emission computerized tomography):ti,ab,kw OR (single-photon emission ct scan):ti,ab,kw OR (single photon emission ct scan):ti,ab,kw (single-photon emission-computed tomography) OR (emission-computed tomography, single-photon):ti,ab,kw OR (single photon emission computed tomography):ti,ab,kw OR (tomography, emission-computed, single-photon):ti,ab,kw OR (ct scan, single-photon emission):ti,ab,kw OR (ct scan, single photon emission):ti,ab,kw (radionuclide tomography, single-photon emission-computed):ti,ab,kw OR (radionuclide tomography, single photon emission computed):ti,ab,kw OR (tomography, single-photon, emission-computed):ti,ab,kw OR (tomography, positron-emission):ti,ab,kw OR (pet scan):ti,ab,kw OR (pet scans):ti,ab,kw OR (scan, pet):ti,ab,kw (pet imaging) OR (imaging, pet):ti,ab,kw OR (pet imagings):ti,ab,kw OR (positron-emission tomography):ti,ab,kw OR (positron emission tomography):ti,ab,kw OR (positron-emission tomography imaging):ti,ab,kw (imaging, positron-emission tomography) OR (positron emission tomography imaging):ti,ab,kw OR (positron-emission tomography imagings):ti,ab,kw OR (tomography imaging, positron-emission):ti,ab,kw |
| **EmBase** | 'parkinson disease'/exp OR 'parkinson disease, idiopathic':ab,ti OR 'idiopathic parkinson disease':ab,ti OR 'lewy body parkinson disease':ab,ti OR 'primary parkinsonism':ab,ti OR 'parkinsonism, primary':ab,ti OR 'lewy body disease':ab,ti OR 'dementia, lewy body':ab,ti OR 'lewy body disease, diffuse':ab,ti OR 'lewy body type senile dementi':ab,ti OR 'lewy body disease, cortical':ab,ti OR 'cortical lewy body disease':ab,ti OR 'lewy body dementia':ab,ti OR 'diffuse lewy body disease':ab,ti AND 'tau proteins':ab,ti OR 'proteins, tau':ab,ti OR 'tau protein':ab,ti OR 'protein, tau':ab,ti OR 'tau':ab,ti OR 'tauopathies':ab,ti OR 'tauopathy':ab,ti AND 'positron-emission tomography':ab,ti OR 'positron emission tomography':ab,ti OR 'positron-emission tomography imaging':ab,ti OR 'imaging, positron-emission tomography':ab,ti OR 'positron emission tomography imaging':ab,ti OR 'positron-emission tomography imagings':ab,ti OR 'tomography imaging, positron-emission':ab,ti OR 'tomography, positron-emission':ab,ti OR 'pet scan':ab,ti OR 'pet scans':ab,ti OR 'scan, pet':ab,ti OR 'pet imaging':ab,ti OR 'imaging, pet':ab,ti OR 'pet imagings':ab,ti OR ‘tomography, emission-computed, single-photon':ab,ti OR 'ct scan, single-photon emission':ab,ti OR 'ct scan, single photon emission':ab,ti OR 'radionuclide tomography, single-photon emission-computed':ab,ti OR 'radionuclide tomography, single photon emission computed':ab,ti OR 'tomography, single-photon, emission-computed':ab,ti OR ‘single-photon emission computerized tomography':ab,ti OR 'single photon emission computerized tomography':ab,ti OR 'single-photon emission ct scan':ab,ti OR 'single photon emission ct scan':ab,ti OR 'single-photon emission-computed tomography':ab,ti OR 'emission-computed tomography, single-photon':ab,ti OR 'single photon emission computed tomography':ab,ti OR 'tomography, single-photon emission-computed':ab,ti OR 'spect':ab,ti OR 'cat scan, single-photon emission':ab,ti OR 'cat scan, single photon emission':ab,ti OR 'single-photon emission computer-assisted tomography':ab,ti OR 'single photon emission computer assisted tomography':ab,ti |
| **Web of Science** | ((TS=(Parkinson Disease OR Idiopathic Parkinson's Disease OR Lewy Body Parkinson's Disease OR Parkinson's Disease, Idiopathic OR Parkinson's Disease, Lewy Body OR Parkinson Disease, Idiopathic OR Parkinson's Disease OR Idiopathic Parkinson Disease OR Lewy Body Parkinson Disease OR Primary Parkinsonism OR Parkinsonism, Primary OR Paralysis Agitans OR PD)) AND TS=(tau Proteins OR Proteins, tau OR tau Protein OR Protein, tau OR tau OR Tauopathies OR Tauopathy)) AND TS=(Positron-Emission Tomography OR Positron Emission Tomography OR Positron-Emission Tomography Imaging OR Imaging, Positron-Emission Tomography OR Positron Emission Tomography Imaging OR Positron-Emission Tomography Imagings OR Tomography Imaging, Positron-Emission OR Tomography, Positron-Emission OR Tomography, Positron Emission OR PET Scan OR PET Scans OR Scan, PET OR PET Imaging OR Imaging, PET OR PET Imagings OR Tomography, Emission-Computed, Single-Photon OR CT Scan, Single-Photon Emission OR CT Scan, Single Photon Emission OR Radionuclide Tomography, Single-Photon Emission-Computed OR Radionuclide Tomography, Single Photon Emission Computed OR Tomography, Single-Photon, Emission-Computed OR Single-Photon Emission Computerized Tomography OR Single Photon Emission Computerized Tomography OR Single-Photon Emission CT Scan OR Single Photon Emission CT Scan OR Single-Photon Emission-Computed Tomography OR Emission-Computed Tomography, Single-Photon OR Single Photon Emission Computed Tomography OR Tomography, Single-Photon Emission-Computed OR SPECT OR CAT Scan, Single-Photon Emission OR CAT Scan, Single Photon Emission OR Single-Photon Emission Computer-Assisted Tomography OR Single Photon Emission Computer Assisted Tomography)  <http://www.webofscience.com/wos/alldb/summary/0b22c540-1aac-4aeb-b22d-2e2c516bf3f9-3d6079d4/relevance/1> |
