# Supplementary material for: Tau-PET imaging in Parkinson's disease: a systematic review and meta-analysis
Source: Front Neurol. 2023 Apr 27;14:1145939. doi: 10.3389/fneur.2023.1145939 (PMC10174250; doi:10.3389/fneur.2023.1145939)
Supplement: Supplementary file 1 [file Data_Sheet_1.ZIP › Supplementary/Supplementary Table 2.docx]

**Supplementary Table 2.** **Quality assessment scores for the included studies.**

|  |  | **Selection** | | | |  | **Comparability** |  | **Exposure** | | |  |
| --- | --- | --- | --- | --- | --- | --- | --- | --- | --- | --- | --- | --- |
|  |  | Definition of the cases | Representativeness of the cases | Selection of Controls | Definition of Controls |  | Comparability of cases and controls on the basis of the design or analysis |  | Ascertainment of exposure | Same method of ascertainment for cases and controls | Non-Response rate | **Total** |
| Kepe | 2013 | * | * |  | * |  | * |  | * | * | * | 7 |
| Cho | 2016 | * | * |  | * |  | * |  | * | * | * | 7 |
| Coakeley | 2016 | * |  |  | * |  | ** |  | * | * | * | 7 |
| Buongiorno | 2016 | * | * |  | * |  | ** |  | * | * | * | 8 |
| Gomperts | 2016 | * | * |  | * |  | * |  | * | * | * | 7 |
| Smith | 2017 | * | * |  | * |  | * |  | * | * | * | 7 |
| Schonhaut | 2017 | * | * |  | * |  | * |  | * | * | * | 7 |
| Hansen | 2017 | * |  |  | * |  | ** |  | * | * | * | 7 |
| Coakeley | 2017 | * |  |  | * |  | ** |  | * | * | * | 7 |
| Lee | 2018 | * | * | * | * |  | ** |  | * | * | * | 9 |
| Ossenkoppele | 2018 | * | * | * | * |  |  |  | * | * | * | 7 |
| Schönecker | 2019 | * | * | * | * |  |  |  | * | * | * | 7 |
| Brendel | 2020 | * | * |  | * |  |  |  | * | * | * | 6 |
| Li, C.H. | 2021 | * | * |  | * |  |  |  | * | * | * | 6 |
| Li, L | 2021 | * | * |  | * |  | ** |  | * | * | * | 8 |
