# Supplementary material for: Tau-PET imaging in Parkinson's disease: a systematic review and meta-analysis
Source: Front Neurol. 2023 Apr 27;14:1145939. doi: 10.3389/fneur.2023.1145939 (PMC10174250; doi:10.3389/fneur.2023.1145939)
Supplement: Supplementary file 1 [file Data_Sheet_1.ZIP › Supplementary/Supplementary Table 3.docx]

**Supplementary Table 3. Sensitivity and publication bias analyses between PD and HCs subjects.**

| Region | Sensitivity | Publication bias | |  | Post Trim-and-Fill model | | | |
| --- | --- | --- | --- | --- | --- | --- | --- | --- |
|  | leave-1-out | T | P |  | Missing studies | SMD [95% CI] | Z | P |
| Global | 3/3 | -1.81 | 0.321 |  | N/A | N/A | N/A | N/A |
| Frontal lobe | 5/5 | 0.39 | 0.722 |  | N/A | N/A | N/A | N/A |
| Prefrontal lobe | 3/3 | 0.75 | 0.592 |  | N/A | N/A | N/A | N/A |
| Parietal lobe | 6/6 | 0.04 | 0.970 |  | N/A | N/A | N/A | N/A |
| Sub.parietal lobe | 2/2 | N/A | N/A |  | N/A | N/A | N/A | N/A |
| Inf.parietal lobe | 2/2 | N/A | N/A |  | N/A | N/A | N/A | N/A |
| Occipital lobe | 4/4 | 1.97 | 0.188 |  | N/A | N/A | N/A | N/A |
| Lat.temporal lobe | 3/3 | -0.08 | 0.948 |  | N/A | N/A | N/A | N/A |
| Med.temporal lobe | 2/2 | N/A | N/A |  | N/A | N/A | N/A | N/A |
| Sup.temporal lobe | 3/3 | 2.38 | 0.253 |  | N/A | N/A | N/A | N/A |
| Mid.temporal lobe | 2/2 | N/A | N/A |  | N/A | N/A | N/A | N/A |
| Inf.temporal lobe | 4/4 | 1.39 | 0.300 |  | N/A | N/A | N/A | N/A |
| Striatum | 2/2 | N/A | N/A |  | N/A | N/A | N/A | N/A |
| Substantia nigra | 6/6 | 0.88 | 0.429 |  | N/A | N/A | N/A | N/A |
| Red nucleus | 2/2 | N/A | N/A |  | N/A | N/A | N/A | N/A |
| Caudate nucleus | 4/4 | 1.72 | 0.227 |  | N/A | N/A | N/A | N/A |
| Subthalamic nucleus | 5/5 | -0.74 | 0.511 |  | N/A | N/A | N/A | N/A |
| Putamen | 6/6 | 0.69 | 0.529 |  | N/A | N/A | N/A | N/A |
| Globus pallidus | 7/7 | 1.35 | 0.235 |  | N/A | N/A | N/A | N/A |
| Dentate nucleus | 5/5 | 0.03 | 0.978 |  | N/A | N/A | N/A | N/A |
| Thalamus | 4/4 | -0.30 | 0.793 |  | N/A | N/A | N/A | N/A |
| Midbrain | 3/3 | -0.62 | 0.646 |  | N/A | N/A | N/A | N/A |
| Pons | 2/2 | N/A | N/A |  | N/A | N/A | N/A | N/A |
| Ant.cingulate | 2/2 | N/A | N/A |  | N/A | N/A | N/A | N/A |
| Post.cingulate | 4/4 | -2.89 | 0.102 |  | N/A | N/A | N/A | N/A |
| Entorhinal | 4/4 | 0.43 | 0.709 |  | N/A | N/A | N/A | N/A |
| Hippocampus | 3/3 | -0.35 | 0.784 |  | N/A | N/A | N/A | N/A |
| Precuneus | 4/4 | 2.40 | 0.139 |  | N/A | N/A | N/A | N/A |
| Sensorimotor | 2/2 | N/A | N/A |  | N/A | N/A | N/A | N/A |
| Insula | 2/2 | N/A | N/A |  | N/A | N/A | N/A | N/A |
| Amygdala | 2/2 | N/A | N/A |  | N/A | N/A | N/A | N/A |

PD, Parkinson's disease; HCs, healthy controls; SMD, Standardized mean difference; CI, confidence interval.
