# Supplementary material for: Tau-PET imaging in Parkinson's disease: a systematic review and meta-analysis
Source: Front Neurol. 2023 Apr 27;14:1145939. doi: 10.3389/fneur.2023.1145939 (PMC10174250; doi:10.3389/fneur.2023.1145939)
Supplement: Supplementary file 1 [file Data_Sheet_1.ZIP › Supplementary/Supplementary Table 5.docx]

**Supplementary Table 5. Sensitivity and publication bias analyses between PDNC and PDCI subjects.**

| Region | Sensitivity | Publication bias | |  | Post Trim-and-Fill model | | | |
| --- | --- | --- | --- | --- | --- | --- | --- | --- |
|  | leave-1-out | T | P |  | Missing studies | SMD [95% CI] | Z | P |
| Entorhinal | 3/3 | 1.34 | 0.408 |  | N/A | N/A | N/A | N/A |
| Hippocampus | 2/2 | N/A | N/A |  | N/A | N/A | N/A | N/A |
| Sup.temporal lobe | 2/2 | N/A | N/A |  | N/A | N/A | N/A | N/A |
| Mid-inf.temporal lobe | 2/2 | N/A | N/A |  | N/A | N/A | N/A | N/A |
| Inf.temporal lobe | 3/3 | -2.21 | 0.271 |  | N/A | N/A | N/A | N/A |
| Precuneus | 3/3 | -5.35 | 0.118 |  | N/A | N/A | N/A | N/A |

PDCI, Parkinson's disease with cognitive impairment; PDNC, Parkinson's disease with normal cognition; SMD, Standardized mean difference; CI, confidence interval.
