# Supplementary material for: Tau-PET imaging in Parkinson's disease: a systematic review and meta-analysis
Source: Front Neurol. 2023 Apr 27;14:1145939. doi: 10.3389/fneur.2023.1145939 (PMC10174250; doi:10.3389/fneur.2023.1145939)
Supplement: Supplementary file 1 [file Data_Sheet_1.ZIP › Supplementary/Supplementary Table 6.docx]

**Supplementary Table 6. Sensitivity and publication bias analyses between PSP and PD subjects.**

| Region | Sensitivity | Publication bias | |  | Post Trim-and-Fill model | | | |
| --- | --- | --- | --- | --- | --- | --- | --- | --- |
|  | leave-1-out | T | P |  | Missing studies | SMD [95% CI] | Z | P |
| Frontal lobe | 4/4 | -0.32 | 0.778 |  | N/A | N/A | N/A | N/A |
| Prefrontal lobe | 2/2 | N/A | N/A |  | N/A | N/A | N/A | N/A |
| Parietal lobe | 5/5 | -0.50 | 0.649 |  | N/A | N/A | N/A | N/A |
| Occipital lobe | 3/3 | -0.94 | 0.518 |  | N/A | N/A | N/A | N/A |
| Temporal lobe | 3/3 | -1.90 | 0.308 |  | N/A | N/A | N/A | N/A |
| Lat.temporal lobe | 2/2 | N/A | N/A |  | N/A | N/A | N/A | N/A |
| Striatum | 3/3 | -1.86 | 0.314 |  | N/A | N/A | N/A | N/A |
| Caudate nucleus | 4/4 | -0.73 | 0.543 |  | N/A | N/A | N/A | N/A |
| Putamen | 6/6 | 0.86 | 0.439 |  | N/A | N/A | N/A | N/A |
| Globus pallidus | 6/6 | 0.01 | 0.994 |  | N/A | N/A | N/A | N/A |
| Thalamus | 4/4 | -1.49 | 0.274 |  | N/A | N/A | N/A | N/A |
| Subthalamic nucleus | 5/5 | -1.62 | 0.204 |  | N/A | N/A | N/A | N/A |
| Midbrain | 3/3 | -2.76 | 0.221 |  | N/A | N/A | N/A | N/A |
| Substantia nigra | 5/5 | -0.51 | 0.646 |  | N/A | N/A | N/A | N/A |
| Red nucleus | 2/2 | N/A | N/A |  | N/A | N/A | N/A | N/A |
| Pons | 2/2 | N/A | N/A |  | N/A | N/A | N/A | N/A |
| Dentate nucleus | 5/5 | 1.01 | 0.388 |  | N/A | N/A | N/A | N/A |
| Cerebellar deep white matter | 2/2 | N/A | N/A |  | N/A | N/A | N/A | N/A |
| Post.cingulate | 2/2 | N/A | N/A |  | N/A | N/A | N/A | N/A |
| Entorhinal | 2/2 | N/A | N/A |  | N/A | N/A | N/A | N/A |
| Inf.temporal lobe | 2/2 | N/A | N/A |  | N/A | N/A | N/A | N/A |

PD, Parkinson's disease; PSP, progressive supranuclear palsy; SMD, Standardized mean difference; CI, confidence interval.
