# Supplementary material for: Tau-PET imaging in Parkinson's disease: a systematic review and meta-analysis
Source: Front Neurol. 2023 Apr 27;14:1145939. doi: 10.3389/fneur.2023.1145939 (PMC10174250; doi:10.3389/fneur.2023.1145939)
Supplement: Supplementary file 1 [file Data_Sheet_1.ZIP › Supplementary/Supplementary Table 8.docx]

**Supplementary Table 8. Sensitivity and publication bias analyses between PD and DLB subjects.**

| Region | Sensitivity | Publication bias | |  | Post Trim-and-Fill model | | | |
| --- | --- | --- | --- | --- | --- | --- | --- | --- |
|  | leave-1-out | T | P |  | Missing studies | SMD [95% CI] | Z | P |
| Frontal lobe | 2/2 | N/A | N/A |  | N/A | N/A | N/A | N/A |
| Occipital lobe | 2/2 | N/A | N/A |  | N/A | N/A | N/A | N/A |
| Parietal lobe | 2/2 | N/A | N/A |  | N/A | N/A | N/A | N/A |
| Inf.temporal lobe | 4/4 | -3.40 | 0.077 |  | N/A | N/A | N/A | N/A |

PD, Parkinson's disease; DLB, dementia with Lewy bodies; SMD, Standardized mean difference; CI, confidence interval.
